# The Role of Carcinine in Signaling at the *Drosophila* Photoreceptor Synapse

**DOI:** 10.1371/journal.pgen.0030206

**Published:** 2007-12-07

**Authors:** Brendan A Gavin, Susan E Arruda, Patrick J Dolph

**Affiliations:** 1 Department of Biology, Dartmouth College, Hanover, New Hampshire, United States of America; 2 Department of Biology, Franklin Pierce College, Rindge, New Hampshire, United States of America; Stanford University School of Medicine, United States of America

## Abstract

The Drosophila melanogaster photoreceptor cell has long served as a model system for researchers focusing on how animal sensory neurons receive information from their surroundings and translate this information into chemical and electrical messages. Electroretinograph (ERG) analysis of *Drosophila* mutants has helped to elucidate some of the genes involved in the visual transduction pathway downstream of the photoreceptor cell, and it is now clear that photoreceptor cell signaling is dependent upon the proper release and recycling of the neurotransmitter histamine. While the neurotransmitter transporters responsible for clearing histamine, and its metabolite carcinine, from the synaptic cleft have remained unknown, a strong candidate for a transporter of either substrate is the uncharacterized inebriated protein. The *inebriated* gene (*ine*) encodes a putative neurotransmitter transporter that has been localized to photoreceptor cells in *Drosophila* and mutations in *ine* result in an abnormal ERG phenotype in *Drosophila*. Loss-of-function mutations in *ebony*, a gene required for the synthesis of carcinine in *Drosophila*, suppress components of the mutant *ine* ERG phenotype, while loss-of-function mutations in *tan*, a gene necessary for the hydrolysis of carcinine in *Drosophila*, have no effect on the ERG phenotype in *ine* mutants. We also show that by feeding wild-type flies carcinine, we can duplicate components of mutant *ine* ERGs. Finally, we demonstrate that treatment with H_3_ receptor agonists or inverse agonists rescue several components of the mutant *ine* ERG phenotype. Here, we provide pharmacological and genetic epistatic evidence that *ine* encodes a carcinine neurotransmitter transporter. We also speculate that the oscillations observed in mutant *ine* ERG traces are the result of the aberrant activity of a putative H_3_ receptor.

## Author Summary

During signaling in the nervous system, individual nerve cells transfer information to one another by a complex process called synaptic transmission. This communication involves the release of a specific neurotransmitter into the synaptic cleft, which then triggers signaling in the downstream neuron by binding to and activating specific cell surface receptors. In order to terminate the neuronal signal, the neurotransmitter must be rapidly removed from the synaptic cleft. This is done by two mechanisms: the neurotransmitter can be degraded or modified, or the transmitter can be taken up by the presynaptic neuron and packaged into vesicles for reuse. In the compound eye of the fruitfly D. melanogaster, the photoreceptor cell responds to light and releases histamine into the synaptic cleft. This signal is terminated by the removal of histamine from the synapse and the enzymatic conversion of histamine to carcinine. We have shown that it is not sufficient just to modify the histamine neurotransmitter, but it is also important to remove carcinine from the photoreceptor synapse. The failure to adequately remove carcinine results in defects in the visual transduction process. Moreover, the work suggests that carcinine itself modulates vision by regulating histamine release into the synapse.

## Introduction

An exceedingly complex regulation is involved in the synthesis, release, activity, and recycling or degradation of neurotransmitter in the nervous system of animal species. This regulation may involve neurotransmitter degradation within the synapse [1], reuptake of neurotransmitter by presynaptic neurons [2], recycling of neurotransmitter by neighboring cells [3], and/or activation of receptors that trigger positive/negative feedback loops resulting in an increase/decrease of neurotransmitter release in presynaptic neurons [4]. Many of the mechanisms and machinery components associated with this regulation have been well conserved across species, ranging from Caenorhabditis elegans to humans [5]. D. melanogaster is often utilized when studying neurotransmitter dynamics because of its malleable genetics, strong phenotypes in the presence of neurotransmission defects, and its sensitivity to numerous neuropharmacological compounds that have been shown to exert similar effects in humans (for review, see [6]).

The major neurotransmitter released by photoreceptor cells in *Drosophila* is histamine [7], which is biosynthesized from histidine by the enzyme histidine decarboxylase (Hdc) found within the photoreceptor cell [8]. Upon excitation by light, the photoreceptor cell depolarizes and releases histamine, which then binds to a postsynaptic histamine-gated chloride channel, resulting in the hyperpolarization of the postsynaptic neuron [9,10]. Each synaptic cartridge is surrounded by three glial cells that invaginate into the photoreceptor terminals by means of fingerlike projections known as capitate structures [11]. While the exact site of histamine reuptake is currently unknown, the histamine remaining in the synaptic cleft is thought to be rapidly taken up by glial cells, possibly at the site of these capitate structures [12]. In glial cells this histamine is converted by the enzyme N-β-alanyl dopamine synthase, encoded by the *ebony* gene in *Drosophila*, into β-alanyl-histamine, also known as carcinine [3,13]. This carcinine is then released by the glial cell as an “inactive” conjugate of histamine, again possibly at the site of capitate structures, where it is then taken up by the presynaptic neuron. Once in the photoreceptor cell, the carcinine is converted by the enzyme N-β-alanyl dopamine hydrolase, encoded by the *tan* gene, back into the original neurotransmitter histamine [13,14]. It is proposed that the combination of histamine biosynthesis through histidine decarboxylase and the recycling of histamine by ebony and tan enzymes defines the total pool of histamine available at the photoreceptor cell synapse. The transporters responsible for histamine uptake by glial cells, and for carcinine internalization by photoreceptor cells, are currently unknown.

The *ine* gene is believed to encode a putative neurotransmitter transporter, and two *ine* cDNAs have been sequenced and identified [15],[16]. The shorter cDNA, *ine-RB*, encodes the protein Ine-P2, while the longer cDNA, *ine-RA*, encodes the protein Ine-P1, which contains an additional ∼300 amino acids at its N terminus. The function of the additional N-terminal region of ine-P1 is currently unknown. Despite efforts to identify the neurotransmitter transported by inebriated in transfected Xenopus laevis oocytes, the substrate of the inebriated protein has remained elusive [17]. Mutations in the *ine* gene result in an increase in the rate of onset of long-term facilitation at the larval neuromuscular junction [18], as well as an increase in the neuronal excitability associated with mutations in the *Shaker* gene, which encodes the α-subunit of a potassium channel [19]. Both of these neuronal excitability phenotypes are believed to be caused by the defective reuptake of an unknown neurotransmitter, and thus the overstimulation of postsynaptic neurons. A third, and less understood, phenotype associated with *ine* mutations is manifested as an aberrant electroretinogram (ERG) [15,20]. ERGs measure the mass retinal response of the eye to a stimulus of light, and the ERG of *ine* mutants is characterized by several defects, including most noticeably a series of strong oscillations in the presence of light [15,20]. Recently, in an excellent and comprehensive review of histaminergic neuronal signaling in arthropods, it was proposed that the *ine* gene in *Drosophila* might encode the carcinine neurotransmitter transporter [21]. Here, we provide genetic and pharmacological evidence linking the mutant *ine-*associated phenotype with the buildup of carcinine in the photoreceptor synaptic cleft and with the activity of a putative H_3_ receptor in the *Drosophila* eye.

## Materials and Methods

### Fly Stocks

The *repo-GAL4*, “long”-*GMR-GAL4*, *w^1118^*, *tan^1^*, *tan^2^*, *e^1^*, *e^11^*, *ort^Pbac^* fly lines were all obtained from Bloomington Stock Center. *Hdc^P218^* and *ort^5^* fly mutants were obtained from W. Pak (Purdue University) while the *UAS-ine-RB* and *ine^2^* transgenic fly lines were obtained from M. Stern (Rice University). All stocks were maintained in constant darkness at room temperature. Flies carrying two or three mutations/transgenes were generated by standard genetic methodologies. All wild-type flies were of the *w^1118^* background.

### Electroretinography

Flies were anesthetized by exposure to carbon dioxide and immobilized within a rotating disc using a drop of molten myristic acid (Akros). To record voltage changes within the eye, an electrode filled with signa gel (Parker Labs) was placed on the surface of the eye, while a second gel-filled electrode was gently inserted into the thorax. For light treatments a halogen lamp controlled by a Model T132 Uniblitz shutter was used. All light treatments, unless otherwise stated, were performed using a 580 nm filter and were 4 s in duration. To reduce the effects that exogenous sources of histamine might have on *ine^2^Hdc^P218^* flies during ERG analysis, these animals were starved for 24 h before testing. To induce depolarization spikes in *ort^5^* flies, two 4-s pulses of 480 nm light, followed by two 4-s pulses of 580 nm of light, were delivered to the flies, and a trace was taken during the second 580 nm pulse for analysis. Voltage changes were amplified using a DAM50 amplifier (World Precision Instruments) recorded using Powerlab 4/30 (AD Instruments, Colorado Springs, CO) and analyzed using Chart 5 software (AD Instruments). Oscillation frequency was determined by counting and averaging the number of repolarization spikes observed within 0.2 s of light exposure in either *ine^2^* or carcinine-treated fly ERG recordings.

### Drug Treatment

Thioperamide, immepip, and histamine were obtained from Sigma and carcinine was obtained from Peninsula Laboratories. All compounds were reconstituted in sterilized water for long-term storage. Flies were treated overnight in vials containing Kimwipes soaked with 200 μl of 1% sucrose solution with or without drug compound. Histamine was delivered to flies at a concentration of 10% [22]. Thioperamide and immepip were used at 0.5% and carcinine at either 5% or 10%. Flies were starved for 24 h before drug treatment.

### Reverse transcriptase PCR

RNA was purified from either embryos or adult fly heads of the *w^1118^* background by employing an RNeasy Mini Kit (Qiagen Sciences). cDNA was generated from purified RNA by utilizing MMLV-Reverse Transcriptase (Fisher Scientific). Forward primers specific to either the *ine-RA* or *ine-RB* transcripts and a reverse primer common to both transcripts were obtained from Integrated DNA Technologies. The *ine-RA* forward primer was ATCGATGGCCACTTCCGGATTACA, the *ine-RB* forward primer was ATCAGTTGCCACTCCCAGTTTCCA, and the reverse primer used to generate PCR product from both transcripts was TATCCTATGCAGGCCAGGACGAAT. Products were generated and amplified by means of PCR using Taq polymerase, buffers obtained from Invitrogen, and a TECHNE, TC-312 thermocycler (Bartoworld Scientific) using the following parameters for 35 cycles (94 °C for 30 s, 55 °C for 30 s, and 72 °C for 90 s). PCR products were separated in a 1% agarose gel and then stained using ethidium bromide.

## Results

### Glial- or Photoreceptor-Specific Expression of Inebriated Protein Rescues ERG Oscillation Phenotype

**Figure 1:**
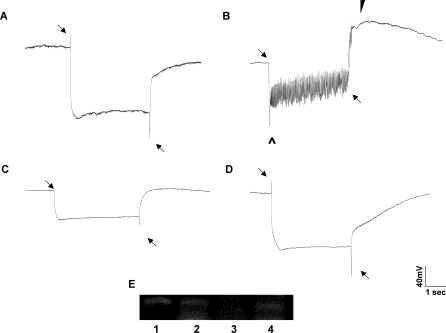
Rescue of *ine^2^*-Associated Oscillations with Expression of INE in Both Glial and Photoreceptor Cells ERG recordings from (A) wild type, (B) *ine^2^*, (C) *ine^2^* expressing the *UAS-ineRB* cDNA in photoreceptors, and (D) *ine^2^* expressing the *UAS-ineRB* cDNA in glial cells. Arrows indicate on and off transients present in (A) wild-type but not (B) *ine^2^* mutant recordings. A sharp depolarization response (unfilled arrowhead), a hyperpolarization (filled arrowhead) response and oscillations are present in (B) *ine^2^* ERG recordings. Note that *ine* expression in either photoreceptor or glial cells results in a rescue of *ine^2^*-associated oscillations, on and off transients (arrows), and the hyperpolarization response. (E) Measurement of *ine-RA* and *ine-RB* mRNA in wild-type embryos or adult heads. 1 = *ine-RA* product from adult heads, 2 = *ine-RA* product from embryos, 3 = *ine-RB* product from adult heads, and 4 = *ine-RB* product from embryos. The *ine-RA* PCR product is 336 bp, while the *ine-RB* product is 338 bp. Note that there is very little *ine-RB* mRNA found in adult heads.

An ERG recording from a wild-type fly (Figure 1A) contains a receptor component, or the depolarization response upon exposure to light, and on and off transient spikes that indicate the response downstream of the photoreceptor cell (arrows, Figure 1A). The ERG of *ine* mutants contains an intact receptor component but with the addition of an initial depolarization spike (unfilled arrowhead, Figure 1B) and prominent oscillations superimposed on the depolarization response (Figure 1B). These oscillations have a wide range of frequencies from 40–90 Hz. These mutants also possess reduced on and off transients (arrows, Figure 1B), indicating impaired photoreceptor synaptic transmission. Finally, these *ine* mutant ERGs often display a hyperpolarization following a light response (arrowhead, Figure 1B). We observed all of these previously described ERG phenotypes when using either *ine^2^* or *ine^3^* allele flies. The *ine^3^* allele is the result of a deletion of the majority of the *ine* open reading frame common to both *ine-RA* and *ine-RB* [16], while the mutation associated with *ine^2^* was identified as being a nonsense mutation in codon 125 of the *ine* gene and is believed to only affect the *ine-RA-*encoded isoform [23]. Because the *ine^3^* allele is associated with reduced viability, and because we could discern no observable difference between the ERG traces of *ine^2^* and *ine^3^* flies, we made use solely of the *ine^2^* fly line for all of our experiments and genetic crosses.

Intracellular voltage recording experiments suggest that the oscillations observed in *ine* mutants originate in the photoreceptor cell, and that they are not the result of synaptic feedback [24]. However, if *ine* does encode a neurotransmitter transporter, its expression and localization are not necessarily restricted to photoreceptor cells, as neurotransmitter transporters often function from neighboring glial cells. Indeed, a previous study demonstrated that expression of *ine* in either neurons or glial cells was sufficient to rescue several mutant *ine*-associated defects at the neuromuscular junction [23]. In order to confirm that the inebriated protein is needed at the photoreceptor cell synapse, we tested whether the *ine^2^* mutant phenotype could be rescued by expressing the *ineRB* transcript in photoreceptor and glial cells. The *UAS-ine-RB* transgenic fly line [23,25] contains the *ine-RB* cDNA under the control of the upstream activator sequence of the yeast Gal4 transcription factor. These *UAS-ine-RB* flies will only express Ine-P2 when crossed with a second line of flies expressing Gal4. The Gal4 lines utilized were “long”-*GMR-GAL4*, which expresses Gal4 protein specifically in photoreceptor cells [26], and *repo-GAL4*, which expresses Gal4 in glial cells [27]. A strong rescue of the *ine^2^* ERG phenotype was observed when *ine-RB* was expressed in either photoreceptor or glial cells (Figure 1C and 1D) compared to non-rescued *ine^2^* or wild-type control (*w^1118^*) ERGs (Figure 1A and 1B). As expected, the *UAS-ine-RB* transgene failed to rescue the *ine^2^* phenotype if neither GAL4 transgene was present (unpublished data). The oscillations observed in ERG traces from these transgenic rescued animals were either absent or greatly reduced, and the hyperpolarization response was also significantly diminished. Finally, the rescued ERG traces contained larger on and off transients than the *ine^2^* non-rescued controls (arrows, Figure 1C and 1D). Expression of *ine-RB* in glial cells appears to give a stronger and more consistent rescue of the *ine^2^* ERG phenotype than when expressed in photoreceptor cells. This may be due to stronger expression of the Gal4 transcription factor in *repo-GAL4* flies than in *GMR-GAL4* animals, or it may be due to the need for full-length *ine-RA*, rather than *ine-RB*, expression in photoreceptor cells. It is also surprising that *ine-RB* expression has the ability to rescue the *ine^2^* ERG phenotype, as the *ine-RB* transcript was previously thought to remain intact in *ine^2^* mutant flies [25]. These results suggest that *ine-RB* is normally expressed at only low levels compared to *ine-RA*, and that overexpression of *ine-RB* is sufficient in compensating for the loss of *ine-RA* associated with *ine^2^* mutants. Reverse transcriptase PCR experiments confirm these suspicions; *ine-RB* is expressed at low levels in adult wild-type heads compared to robust expression of *ine-RB* in the developing embryo (Figure 1E). The *ine-RA* transcript was found at high levels in both wild-type embryos and adult heads (Figure 1E).

### Histamine Is Required for the *ine* Mutant ERG Phenotype

**Figure 2:**
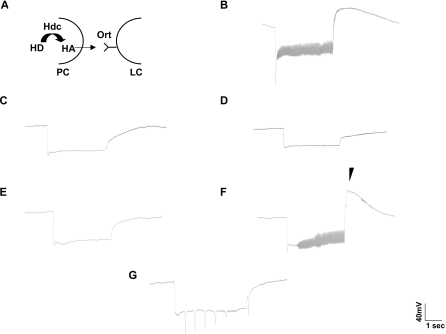
Histamine Synthesis, But Not Postsynaptic Histamine Signaling, Is Required for the *ine* Mutant ERG Phenotype (A) Simplistic diagram showing the synthesis and activity of histamine in the *Drosophila* eye. HD, histidine; Hdc, histidine decarboxylase; HA, histamine; Ort, histamine-gated chloride channel; PC, photoreceptor cell; LC, postsynaptic laminar cell. Histamine is generated by histidine decarboxylase from histidine in the photoreceptor cell. It is then released and acts upon a histamine-gated chloride channel on the postsynaptic laminar cell to trigger downstream signaling in the eye. ERG recordings from (B) *ine^2^*, (C) *Hdc^P218^*, (D) *ine^2^Hdc^P218^*, (E) *ort^5^*, and (F) *ine^2^;ort^5^* flies. Note that *Hdc^P218^* rescues the *ine^2^*-oscillations, but that *ort* mutations do not. (G) ERG recordings from 76% of *ort^5^* flies exhibit strong depolarization spikes. Filled arrowheads indicate a hyper-repolarization response. All flies possessed white eyes due to the presence of the *w^1118^* mutation.

The ability to rescue the *ine^2^* ERG response by expressing inebriated protein in photoreceptor and glial cells suggests that inebriated functions primarily at the site of the photoreceptor cell in the eye. Histamine is believed to be the predominant neurotransmitter that signals between photoreceptor cells and second order laminar neurons in the optic lobe [28], and it is possible that inebriated serves as a histamine transporter. However, previous studies showed that when inebriated protein from the tobacco hornworm *Manduca sexta,* which has significant homology to the inebriated protein from *Drosophila*, was expressed in Xenopus laevis eggs, it was unable to transport histamine across the cell membrane [17]. However, these authors do propose that a second unknown protein may be required to assist inebriated in proper neurotransmitter transport function, or that inebriated may possess different substrates in *Manduca* compared to *Drosophila*. Thus, histamine could still be the substrate of inebriated in *Drosophila*. Histamine is generated by the activity of histidine decarboxylase, encoded by the *Hdc* gene in *Drosophila* (Figure 2A). Mutations in the *Hdc* gene, such as in the case of the *Hdc^P218^* allele, result in flies possessing disrupted photoreceptor synaptic transmission, as demonstrated by the lack of on and off transients in their ERGs ([8], and Figure 2C). Approximately 80% of flies that were homozygous for both the *Hdc^P218^* and *ine^2^* alleles displayed ERGs with no oscillations (Figure 2D) when compared to *ine^2^* controls (Figure 2B). There was a small percentage (∼20%) of *Hdc^P218^ ine^2^* flies that displayed weak or delayed oscillations. However, the ERGs from these flies that displayed this weak rescue also possessed on and off transients, suggesting that the *Hdc^P218^* allele was either not fully penetrant in these double mutants, or that their food provided an outside source of histamine. This is not surprising, as *Drosophila* photoreceptors are known to regain some function from exogenous histamine taken up at minute levels in their food [22]. The ERGs from *Hdc^P218^ ine^2^* also often lack the hyperpolarization response characteristic of *ine^2^* flies (Figure 2D). These findings suggest that histamine production or signaling plays a strong role in the oscillation and hyperpolarization phenotype observed in *ine^2^* traces.

The postsynaptic receptor for histamine in *Drosophila* is a histamine-gated chloride channel (Figure 2A), and a subunit of this channel is encoded by the *ora transientless* (*ort*) gene [10]. The *ort^5^* and *ort^Pbac^* alleles both result in reduced activity of this histamine receptor in *Drosophila*, as shown by the lack of on and off transients in their ERG traces ([10] and Figure 2E). If *ine* encodes a histamine neurotransmitter transporter, then reduced function of this protein may result in an excess of histamine in the synaptic cleft, and this excess of neurotransmitter may be acting upon this postsynaptic histamine receptor to somehow generate the observed oscillations. If this were the case, then *ine^2^*;*ort* double mutants should have reduced oscillations. However, neither *ine^2^;ort^5^* (Figure 2F) nor the *ine^2^;ort^Pbac^* (unpublished data) double mutants exhibited rescue of the oscillation or hyperpolarization components of the *ine^2^* ERG recordings, indicating that the oscillations do not arise from histamine signaling in downstream neurons. Moreover, since *ort* mutations block signaling in laminar neurons, these data are consistent with the oscillations being generated in the photoreceptor cells. Surprisingly, the *ort^5^* allele, which is the result of a frameshift mutation and therefore likely serves as a null allele for this gene [10], often displays strong depolarization spikes of its own in the receptor component of its ERG trace (Figure 2G). Therefore, mutations in *Hdc*, which block the formation of histamine, rescue *ine^2^* whereas mutations in *ort*, which still allow for the synthesis of histamine, fail to rescue.

### *Ebony* Activity Is Required for the *ine* Mutant ERG Phenotype

**Figure 3:**
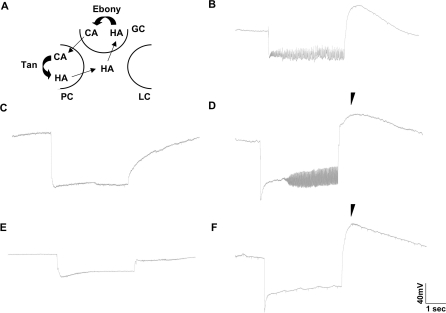
Ebony, But Not Tan, Activity Is Required for the *ine* Mutant ERG Phenotype (A) Simplistic diagram showing the recycling of histamine in the *Drosophila* eye. HA, histamine; CA, carcinine; PC, photoreceptor cell; LC, postsynaptic laminar cell, and GC, glial cell. Histamine is taken up from the synaptic cleft by glial cells where it is converted by ebony into carcinine. Carcinine is then shipped to photoreceptor cells where it is converted back to histamine by the enzyme tan. ERG recordings from (B) *ine^2^*, (C) *tan,* (D) *tan^1^;ine^2^*, (E) *ebony^11^*, and (F) *ine^2^;ebony^11^* flies. Note that *ine^2^* oscillations are rescued by mutations in *ebony* but not in *tan.*

The ablation of the *ine* mutant ERG phenotype upon the introduction of *Hdc*, but not *ort*, mutations, suggests that histamine is involved in generating the *ine^2^* ERG phenotype, but that histamine's downstream signaling in the optic lobe is not. The recycling pathway of histamine in the eye has been well elucidated [13,14,29,30]. It has been shown that following release into the synaptic cleft, histamine is rapidly taken up by neighboring glial cells and is converted by the β-alanyl-dopamine synthase, encoded by the gene *ebony*, into β-alanyl-histamine, also known as carcinine (Figure 3A). This carcinine is then transported into the presynaptic photoreceptor cell and is converted back into histamine by β-alanyl-dopamine-hydrolase, encoded by the gene *tan*, for use as a recycled source of neurotransmitter (Figure 3A). Both *tan* and *ebony* mutations in *Drosophila* are associated with significant reductions in size of the on and off transients in ERG traces (Figure 3C and 3E), due to the loss of this recycled pool of histamine in the eye. Introduction of either *tan^2^* (unpublished data) or *tan^1^* mutations into an *ine^2^* background failed to have any effect in reducing the size of the oscillations or hyperpolarization response when compared to *ine^2^* mutants alone (Compare Figure 3B with Figure 3D). However, *ine^2^;ebony^1^* (unpublished data) or *ine^2^;ebony^11^* double mutants displayed complete rescue of the oscillation phenotype in all flies tested (Compare Figure 3B with Figure 3F). These data, combined with the fact that histamine synthesis is necessary for the presentation of a mutant ERG phenotype in *ine^2^* flies, provide genetic evidence that carcinine is involved in generating *ine^2^*-associated oscillations.

### Treatment of Wild-type and *ebony* Flies with Carcinine Results in ERG Abnormalities

**Figure 4:**
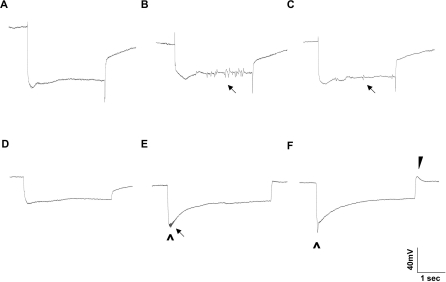
Carcinine Treatment Induces an *ine^2^*-Like ERG Phenotype in Wild-Type and *ebony^11^* Flies ERG recordings from untreated (A) wild-type and (D) *ebony^11^* flies and 5% carcinine-treated (B, C) wild-type and (D, E) *ebony^11^* flies. Note that while untreated wild-type and *ebony^11^* ERG recordings lack oscillations and a hyperpolarization response in response to light, ERG recordings from wild-type flies treated with 5% carcinine overnight display weak oscillations (arrows) in ∼35% of tested animals and ERG recordings from *ebony^11^* flies treated with 5% carcinine exhibit depolarization spikes in response to light (unfilled arrowhead), as well as weak oscillations (arrow) and a hyper-repolarization upon the termination of light (arrowhead).

If *ine* encodes a carcinine neurotransmitter transporter, as the genetic evidence above suggests, than a potential cause of the aberrant ERG phenotypes seen in *ine* mutants could be the buildup of carcinine within the photoreceptor synaptic cleft. In order to test whether or not carcinine is able to induce an *ine^2^*-like ERG phenotype in wild-type animals, *w^1118^* flies were treated with 5% carcinine overnight and then subjected to ERG analysis. Approximately 35% of the *w^1118^* flies treated with carcinine displayed occasional weak oscillations or brief depolarization/repolarization spikes in the photoreceptor response of their ERG traces (Figure 4B and 4C, compare to Figure 4A). While these spikes exhibit no consistent frequency, unlike the oscillations seen in *ine^2^* recordings, the carcinine-induced ERG disturbances were never observed in untreated starved flies. If carcinine was delivered to *ebony^11^* flies, which lack the ability to synthesize carcinine from histamine, they surprisingly displayed an abnormal ERG trace. All *ebony^11^* flies treated with carcinine manifested phenotypes reminiscent of those seen in *ine^2^* ERG traces, including sharp depolarization spikes in response to a light response, weak oscillations, and a hyperpolarization peak upon the termination of light (Figure 4E and 4F, compare to Figure 4D). The oscillations observed in carcinine-treated *ebony^11^* mutants, while only appearing briefly during the initiation of light exposure, were seen at a similar frequency as those found in *ine^2^* ERG recordings (63 spikes/s). As discussed below, a possible mechanism underlying these carcinine-induced ERG disturbances may involve the sensitization of a putative histamine/carcinine receptor.

### Pharmacological Rescue of the *ine^2^* Mutant ERG Phenotype

If inebriated does serve as a carcinine transporter, and if carcinine is indeed building up within the synaptic cleft in *ine^2^* mutants, then this uncleared carcinine appears to somehow be acting on some synaptic receptor to initiate this aberrant oscillation phenotype. The *ine^2^;ort^5^* experiments suggest that this receptor is not the post-synaptic histamine-gated chloride channel. In mammals and various other vertebrate systems, presynaptic histaminergic neurons often contain their own histamine receptors, known as H_3_ receptors. The H_3_ receptor is a G-protein–coupled receptor that was first identified in 1983 by Arrang et al. [4] and is now known to act as a presynaptic autoreceptor that inhibits histamine release from histaminergic neurons in the brain (for review, see [31]). Thus, H_3_ receptors serve to negatively regulate histamine release and synthesis in the presence of high histamine levels in the synaptic cleft. While no H_3_ receptor has been identified yet in *Drosophila*, there are several candidate genes that may encode such a putative receptor. There are numerous well-characterized pharmaceutical compounds that act as agonists, antagonists, or inverse agonists of the H_3_ receptor in vivo in mammals, and recently carcinine was identified as being an inverse agonist of this receptor in mice [32]. It was shown that, rather than reduce histamine release, as occurs in the case of histamine binding to an H_3_ receptor, carcinine had the opposite effect and induced both histamine synthesis and release from presynaptic histaminergic neurons in vivo.

**Figure 5:**
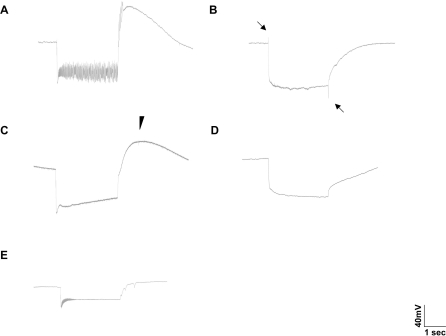
Thioperamide and Immepip Treatment Rescues Oscillations in *ine^2^* ERGs and Ablates Transients in *w^1118^* Flies ERG recordings from untreated (A) *ine^2^* and (B) *w^1118^* and 0.5% thioperamide-treated (C) *ine^2^* and (D) *w^1118^*, and (E) 0.5% immepip-treated *ine^2^* flies. Note that while ERG recordings from untreated *ine^2^* mutants display oscillations and a hyperpolarization response, treatment of *ine^2^* flies overnight with 0.5% thioperamide results in a loss of oscillations and on and off transients but not the hyperpolarization response (arrowhead). (E) Treatment of *ine^2^* flies overnight with 0.5% immepip results in a loss of oscillations and hyperpolarization response in >50% of *ine^2^* mutants tested.

A possible scenario to explain the oscillations seen in *ine^2^* ERGs is that histamine and uncleared carcinine are competing for binding to putative H_3_ receptors, resulting in opposing signaling cascade responses in the photoreceptor cell. If this is the case, disrupting this balance of histamine and carcinine binding to the putative H_3_ receptor in *ine^2^* fly eyes should result in a rescue of ERG oscillations. Indeed, treatment of *ine^2^* flies with 10% carcinine resulted in a rescue of oscillations in 35% of flies (unpublished data), and treatment of *ine^2^* flies with 0.5% thioperamide, another well-characterized and potent inverse agonist of the H_3_ receptor in mammals, resulted in the consistent and complete ablation of oscillations in ERG traces in all flies tested (Compare Figure 5A with Figure 5C). In addition, treatment of *ort^5^* flies with 0.5% thioperamide resulted in a loss of *ort^5^*-associated depolarization spikes (unpublished data). Surprisingly, treatment of wild-type control flies with 0.5% thioperamide resulted in the loss of on and off transients in their ERGs (compare Figure 5B with Figure 5D).

It should also be possible to disrupt the hypothetical balance of histamine and carcinine binding to a photoreceptor cell-specific H_3_ receptor in *ine^2^* by introducing an H_3_ receptor agonist, such as histamine itself. Indeed, treatment of *ine^2^* flies with 10% histamine (unpublished data) or 0.5% immepip (Figure 5E), another potent H_3_ receptor agonist, resulted in a strong rescue of oscillations in >50% of flies tested. Occasionally, weak oscillations and depolarization spikes were still observed in immepip- or histamine-treated *ine^2^* flies (Figure 5E). Neither histamine nor immepip treatment had a strong or consistent effect on the on and off transients seen in wild-type control ERGs. Since immepip and thioperamide are known to be specific agonists and inverse agonists of the mammalian H_3_ receptor, these pharmacological experiments suggest that an H_3_ receptor may exist in *Drosophila* and that abnormal stimulation of this H_3_ receptor is occurring in the eyes of *ine^2^ Drosophila* mutants.

## Discussion

Our findings indicate that the presumed neurotransmitter transporter encoded by the *ine* gene in *Drosophila* transports the histamine metabolite carcinine. We show using genetic epistasis that the oscillations observed in mutant *ine* ERGs require histidine decarboxylase activity and the carcinine-synthesizing enzyme ebony, but not the carcinine-hydrolyzing enzyme tan. We also reveal that treating wild-type flies with carcinine can phenocopy components of the mutant *ine* ERG phenotype. Finally, by rescuing the *ine^2^*-associated phenotype with drugs that target the mammalian H_3_ receptor, we provide pharmacological evidence for the presence of a putative H_3_ receptor in *Drosophila* that may be responsible for the ERG oscillations observed in flies carrying mutations in the *ine* gene.

### The Inebriated Protein Functions in Photoreceptor and Glial Cells

Previous studies involving intracellular voltage recordings of *ine* mutants led the authors to conclude that the oscillations observed in *ine* mutant ERGs were the result of a defect occurring within the photoreceptor cell [24]. We were able to support these conclusions by expressing *ine* specifically in photoreceptor cells and demonstrating a rescue of the *ine^2^*-associated oscillations. Neurotransmitter transporters are often able to function from either the presynaptic neuron or from neighboring glial cells, as shown at the neuromuscular junction in *ine* mutants [23]. We found that glial cell–specific expression of the *ine* gene in *ine^2^* flies resulted in a complete rescue of the *ine* mutant ERG phenotype. It was somewhat unexpected that *ine* expression in glial cells rescued the *ine^2^* phenotypes, as glial cells have been shown to lack tan protein and thus would be unable to convert carcinine back to a recycled pool of histamine [30]. However, it is possible that glial cells do express trace amounts of the enzyme tan to hydrolyze carcinine and generate a renewable source of histamine for photoreceptor cells, and it is also possible that the inebriated protein is expressed in a non-autonomous manner and can be transported from glial cells to photoreceptors in the fly eye.

The finding that an ERG recording can exhibit oscillations is somewhat surprising. An ERG does not record the electrical response of a single photoreceptor, but rather is a collective measure of the retinal photoresponse. Thus, if the mutant *ine*-associated ERG defects are indeed localized to the photoreceptor synapse, as our data and that of previous labs suggest, then one would expect that different photoreceptors would be excited/inhibited at different timepoints, ultimately resulting in the oscillations simply canceling themselves out. The fact that oscillations are indeed observed, and appear to be due to a defect occurring at the photoreceptor synapse, implies the existence of an uncharacterized and complex synchronization of photoreceptor cell de-/repolarization.

### A Postsynaptic Buildup of Carcinine in the *Drosophila* Eye Causes the *ine^2^*-associated Mutant ERG Phenotype

**Figure 6:**
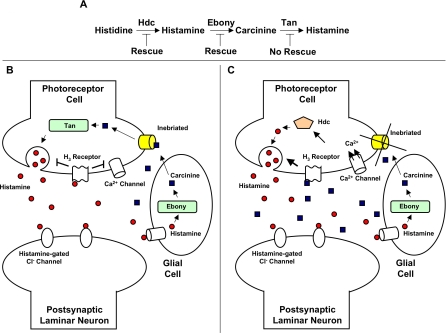
Possible Role of Inebriated in Retinal Signal Transduction in *Drosophila* (A) Epistatic diagram illustrating how mutations in either *Hdc* or *ebony*, but not *tan*, rescue *ine^2^*-associated oscillations. (B) Model of histamine/carcinine dynamics in a wild-type *Drosophila* eye. In a wild-type fly eye, the photoreceptor cell depolarizes in response to light, resulting in a release of histamine into the synaptic cleft. This histamine then binds to and activates postsynaptic histamine-gated chloride channels on laminar neurons, thereby perpetuating the light-induced signaling cascade in the eye. Excess histamine can bind to a putative presynaptic H_3_ receptor resulting in the inhibition of calcium influx and further release of neurotransmitter. Eventually the majority of the histamine is removed from the synaptic cleft by glial cells, where it is converted into carcinine by the enzyme ebony. Carcinine is then taken up by photoreceptor cells, by means of the inebriated neurotransmitter transporter, where it is converted back into histamine by the enzyme tan. (C) Model of histamine/carcinine dynamics in an *ine*-mutant *Drosophila* eye. In *ine* mutants the release of histamine by photoreceptor cells, the uptake of histamine by glial cells, and the conversion of histamine into carcinine are all unaffected. However, the removal of carcinine from the synaptic cleft is presumed defective. This results in an excess of carcinine in the synaptic cleft, which then binds to the putative H_3_ receptor permitting calcium entry, ultimately stimulating the production and release of histamine from the photoreceptor cell. The newly released histamine and excess carcinine compete for binding to the H_3_ receptor, resulting in a fluctuation between inhibition and liberation of calcium channels, ultimately producing the repolarization/depolarization responses that collectively contribute to the observed oscillations seen in *ine* mutant ERGs.

The lack of rescue of *ine^2^*-associated oscillations in flies carrying additional mutations in the postsynaptic histamine receptor gene *ort*, the finding that mutant *ine* oscillations were detected within single photoreceptor cells [24], and our observations that the mutant *ine* phenotype can be rescued when *ine* is expressed in photoreceptors, all combine to strongly suggest that the oscillation phenotype is likely a result of a defect occurring within the photoreceptor itself. In addition, by crossing *ine^2^* animals with *Hdc^P218^* flies, we demonstrated that the *ine^2^-*associated oscillations are dependent upon histamine synthesis. All of these results indicate that histamine is somehow contributing to the aberrant ERG witnessed in *ine^2^* flies, and that histamine appears to be acting on the presynaptic photoreceptor cell to induce this oscillation phenotype. Further epistatic analyses also revealed that ebony, but not tan, activity is required for the generation of oscillations in *ine^2^* ERGs (Figure 6A). These genetic experiments are consistent with *ine* encoding either a carcinine importer found in the photoreceptor cell or a carcinine exporter found in glial cells. The homology of inebriated with other known Na^+^/Cl^−^ neurotransmitter transporters (which import neurotransmitter into cells) [16] suggests that inebriated protein is transporting carcinine into the photoreceptor, and not out of glial cells.

While ebony is known to act on multiple substrates, such as dopamine to generate β-alanyl-dopamine [13], the requirement of histamine synthesis for the maintenance of *ine^2^*-associated oscillations suggests that it is β-alanyl-histamine, or carcinine, that is somehow responsible for the oscillations observed in *ine^2^* ERGs. It should be noted, however, that *ebony* mutations were not sufficient in rescuing the hyperpolarization response observed in mutant *ine* ERG traces (Figure 3F). The origins of this hyperpolarization response are still unclear and further research will be required to elucidate its exact meaning. In *tan* mutants, one would predict that there would be a buildup of carcinine. However, this buildup does not give rise to an ERG recording similar to that of *ine^2^*. This is due most likely to the presence of functional inebriated protein in *tan* mutant flies, which should effectively clear the carcinine from the synaptic cleft for degradation within the photoreceptor cell.

By treating wild-type and *ebony^11^* flies with carcinine and subsequently inducing components of the *ine^2^*-ERG phenotype, we provide further evidence suggesting that the sharp depolarization spike, the oscillations, and the hyperpolarization response all seen in *ine^2^*-ERGs are due to a buildup of carcinine within the photoreceptor synaptic cleft. While the oscillations observed in carcinine-treated wild-type flies do not mimic exactly the oscillations seen in *ine^2^* ERG recordings, it is presumably difficult to replicate the carcinine and histamine balance occurring in the eyes of *ine^2^* animals. Indeed, treatment of wild-type flies with higher (10%) or lower (1%) concentrations of carcinine were less effective in inducing the oscillations than the described 5% carcinine dose (unpublished data).

It is possible that carcinine is being degraded or modified by the fly before the compound is able to exert its effects at the photoreceptor cell. In order to eliminate the activity of one enzyme known to be involved in carcinine metabolism, *tan^1^* flies were treated with 5% carcinine overnight. Surprisingly, none of the *tan^1^* flies treated with carcinine showed an aberrant ERG phenotype (unpublished data). It was surprising that carcinine treatment had a strong effect in flies of the *ebony^11^*, but not the *tan^1^*, background. While the results of these *tan^1^* and *ebony^11^* carcinine-treatment experiments are unexpected, one possible explanation may involve the regulation of carcinine clearance/degradation. The *tan^1^* flies presumably suffer from a perpetual excess of carcinine even before exogenous carcinine treatment, and these flies, in order to reduce their sensitivity to this compound, may consequently decrease the levels of a putative carcinine receptor, increase their rate of carcinine degradation, or increase the levels of inebriated protein for carcinine clearance. However, *ebony^11^* flies are relatively “naïve” to the effects of carcinine, as their ability to synthesize this compound has been greatly diminished, and as a result these animals may have an increased level of the supposed carcinine receptor, a decrease in inebriated receptor levels or a decrease in carcinine degradation, ultimately making them more sensitive to the effects of carcinine treatment.

It remains to be seen whether or not all of the mutant *ine*-associated phenotypes, including increased neuronal excitability [19],[23] and increased sensitivity to osmotic stress [25], are due to the inability of these flies to transport carcinine. It is possible that the inebriated protein transports other compounds that perhaps share the common feature of β-alanine conjugation. This might help explain why none of the more common neurotransmitters were taken up by *ine*-transfected *Xenopus* oocytes [17]. In order to assist in confirming that inebriated is indeed a carcinine neurotransmitter transporter, in vitro experiments, such as neurotransmitter uptake assays, will need to be performed. In addition, the ability of inebriated protein to take up other β-alanyl-neurotransmitters/osmolytes also should be examined.

### What Are the *ine^2^*-Associated Oscillations?

The oscillations present within the photoreceptor response of *ine^2^* ERGs appear as sharp depolarization/repolarization spikes, and this oscillation phenotype is dependent upon both histamine synthesis and ebony activity (Figure 6A), and is sensitive to drugs that target mammalian H_3_ receptors. It is perplexing that the synthesis of a single metabolite, carcinine, could be responsible for both the depolarization and repolarization spikes observed within *ine* mutant ERGs. We speculate that these oscillations are the result of aberrant signaling involving both carcinine and histamine at a putative H_3_ receptor in *Drosophila* (Figure 6B and 6C). H_3_ receptors are an unusual example of the G-protein coupled receptor family, in that they have partial constitutive activity, resulting in a constant small percentage of stimulated G-proteins [33] that trigger a reduction of histamine synthesis and release [34] as well as a decrease in extracellular calcium inflow [35,36,37]. The presence of an H_3_ receptor agonist, such as histamine, causes an increase in activity of the associated G-protein and therefore a stronger inhibition of both histamine release and calcium inflow. Thus, synaptic histamine serves as a negative regulator for its own release and induces a slight repolarization of a stimulated presynaptic histaminergic neuron by inhibiting presynaptic calcium channels. An H_3_ receptor inverse agonist is believed to act by blocking the constitutive activity of the H_3_ receptor, resulting in the liberation from a histamine release checkpoint as well as the release of restrictions on calcium inflow [38]. Recently, it has been shown that carcinine has the ability to act as an inverse agonist of presynaptic H_3_ receptors in mice [32]. While significant further research is required to confirm this hypothesis, we surmise that histamine and carcinine are exerting opposing effects on the polarization state of the histaminergic photoreceptor cell by activating or inhibiting presynaptic calcium channels via a putative *Drosophila* H_3_ receptor. While a recent search of the *Drosophila* genome did not uncover any direct homologs to vertebrate metabotropic histamine receptors [39], the CG7918 gene was listed as a possible candidate for encoding such a receptor, and this gene bears strong homology to genes encoding H_3_ receptors in mammals. In addition, the *ine^2^*-associated oscillations display sensitivity to mammalian H_3_ receptor agonists and inverse agonists, strengthening the possibility that an H_3_ receptor does exist in *Drosophila*. It is still unclear what the origins of the thioperamide-sensitive depolarization spikes are that are observed in *ort^5^* ERGs. The presence of these thioperamide-sensitive spikes in *ort^5^* ERG recordings implies the requirement of some postsynaptic retrograde signal for ERG stability, and this *ort*-dependent signal may be involved in the sensitization of the putative H_3_ receptor.

It was unexpected that thioperamide treatment of wild-type flies resulted in the loss of on and off transients within their ERG traces. It is possible that histamine release was so extreme in the presence of the potent thioperamide that histamine levels were nearly depleted in the eye, resulting in the disruption of downstream signaling events. Indeed, treatment of mice with high concentrations of carcinine, which acts as an inverse agonist of H_3_ receptors similar to thioperamide, was shown to result in significantly lower overall levels of histamine within the brains of treated mice [32]. This model of indirect histamine depletion has also been postulated to occur in *ebony* mutant flies. The absence of on and off transients in *ebony* mutant ERG recordings is attributed to the normal release of histamine by photoreceptor cells, but this histamine subsequently lacks the ability to be “trapped” by β-alanine conjugation, ultimately resulting in histamine diffusing away from the eye [13]. Interestingly, expression of pertussis toxin in photoreceptor and laminar neurons in *Drosophila* results in a similar loss of on and off transients in ERG traces, and this is believed to be the result of inactivation of an unknown G-protein coupled receptor found in photoreceptor cells that is unlikely to be rhodopsin [40]. It is possible that pertussis toxin was acting within photoreceptor cells upon the putative H_3_ receptor in this study, resulting in a lack of negative feedback on histamine synthesis/release, eventually causing the exhaustion/depletion of histamine pools. Further research will be required to confirm or dismiss the presence of a histamine/carcinine-sensitive H_3_ receptor in *Drosophila* photoreceptor cells.

## Supporting Information

### Accession Numbers

The National Center for Biotechnology Information (NCBI) Entrez (http://www.ncbi.nlm.nih.gov/sites/gquery) accession numbers for the genes discussed in this paper are *ebony*, 42521; *Hdc*, 36076; *ine*, 33659; *ort*, 54910; and *tan*, 4478.

## Abbreviations

ERG: electroretinograph
